# GME-Init: Gamma-Moment Equalization for LoRA Initialization in Parameter-Efficient Fine-Tuning

**DOI:** 10.3390/e28080873

**Published:** 2026-08-03

**Authors:** Yuhui Lin, Chaopeng Li, Zhiwei Shen, Jianfeng Liu, Miao Zeng

**Affiliations:** 1 School of Ocean Information Engineering, Jimei University, Xiamen 361021, China; yuhuilin@jmu.edu.cn (Y.L.); shenzhiwei@jmu.edu.cn (Z.S.);; 2 Fujian Provincial Key Laboratory of Oceanic Information Perception and Intelligent Processing, Jimei University, Xiamen 361021, China

**Keywords:** parameter-efficient fine-tuning, LoRA, data-aware initialization, output-moment calibration, skewness, asymmetric initialization, GLUE, remote sensing vision–language models

## Abstract

Low-Rank Adaptation (LoRA) is a representative parameter-efficient fine-tuning method that reduces computational and memory costs without modifying the model architecture. Standard LoRA initializes matrix A from a symmetric distribution, such as Gaussian or Kaiming initialization, and matrix B to zero. Although this provides a statistically neutral starting point, it ignores the influence of task-specific input features on initialization. We propose Gamma-Moment Equalization Initialization (GME-Init), a data-aware asymmetric LoRA initialization method based on output-moment calibration. Using a small calibration set, GME-Init estimates the variance and skewness of target-layer outputs and adjusts the layer-wise initialization scale and asymmetry of LoRA weights, improving their statistical alignment with task-specific skewed representations. GME-Init operates only during initialization and does not change the LoRA architecture, trainable parameter count, training budget, or inference cost. We evaluate it on a GLUE subset with RoBERTa-base, integrate it with AdaLoRA and DoRA, and test it on VRSBench-VQA, VRSBench-Caption, and UCM-Caption using Qwen2.5-VL-3B-Instruct. Results show that GME-Init serves as a simple plug-in PEFT initialization module with no additional inference cost and that it consistently improves standard LoRA and selected LoRA-style methods across the evaluated text understanding and multimodal tasks.

## 1. Introduction

Large pretrained models are increasingly used for text understanding, multimodal question answering, image captioning, and domain-specific information extraction. As model size grows, full-parameter fine-tuning requires substantial memory, computation, and storage for each downstream task. These demands also complicate model deployment and maintenance. Parameter-efficient fine-tuning (PEFT) has become an important approach to adapting pretrained models [1]. Among PEFT methods, Low-Rank Adaptation (LoRA) freezes the pretrained weight matrix and trains only a low-rank update branch. This design reduces fine-tuning costs while largely preserving the original architecture and workflow [2,3].

The initialization state of LoRA directly affects early adapter outputs, gradient directions, and optimization trajectories. Standard LoRA usually initializes matrix *A* from a symmetric distribution, such as a Gaussian or Kaiming distribution, and sets matrix *B* to zero. The initial low-rank update then satisfies ΔW=BA=0. This design avoids perturbing the pretrained model before training, and centering the sampled entries of *A* around zero provides a directionally unbiased statistical starting point. However, this initialization does not reflect the influence of the input feature distribution on the initial statistical state of LoRA. After task inputs pass through the pretrained model and its nonlinear transformations, target-layer outputs may exhibit task-dependent scales and skewed distributions. Therefore, a fixed symmetric initialization with an exactly zero low-rank update may be statistically mismatched with the representations induced by the current task data.

Recent LoRA initialization methods have improved the initial low-rank subspace using pretrained-weight decomposition, downstream activations, input covariance, or gradient information [4,5,6,7,8,9,10]. Although these strategies make initialization more structured or task-aware, they do not directly control whether different target layers begin fine-tuning with compatible output scales and distributional asymmetry. In particular, the joint alignment of output variance and skewness remains insufficiently explored.

This paper revisits LoRA initialization from the perspective of layer-output statistics.

PiSSA and OLoRA construct low-rank initialization subspaces from the geometric structure of pretrained weights. Existing data-aware methods mainly use activations, covariance, constraints, or gradients to identify informative directions in parameter space. In contrast, GME-Init formulates initialization directly in the space of observable layer-output moments. Its central novelty is not a particular sampling distribution; instead, it treats LoRA initialization as a reduced-order calibration problem and solves for layer-specific scale and asymmetry corrections from measured output responses.

A small calibration set estimates the variance and skewness produced by the current input distribution at the target adapter layers. It does not introduce an additional supervised training signal. Based on these statistics, we formulate LoRA initialization as a local calibration problem in output-moment space. We first estimate the second- and third-order moments of target-layer outputs. Coordinated probes then measure effective layer-wise responses and yield approximate scale and asymmetry corrections. Finally, we convert these corrections into concrete low-rank matrix initializations. This process changes only the initial state of the low-rank branch while leaving the LoRA structure, trainable parameter count, rank, training budget, and inference pipeline unchanged.

Following this idea, we propose Gamma-Moment Equalization Initialization (GME-Init). GME-Init estimates layer-output moments through gradient-free calibration passes, then uses coordinated probes to construct effective local response matrices. These matrices describe how the variance and skewness observed at each layer respond to controlled changes in the LoRA initialization scale and asymmetry. GME-Init initializes *A* with a centered Gamma distribution and *B* with a tiny non-zero Gaussian distribution. The non-zero perturbation allows output-moment calibration to take effect, while its controlled magnitude avoids disrupting pretrained representations. GME-Init can also serve as a plug-in initialization module for LoRA-style PEFT variants such as AdaLoRA and DoRA.

The main contributions of this paper are as follows. We identify a limitation of standard LoRA, namely, that the symmetric initialization of *A* and zero initialization of *B* do not reflect how the input feature distribution affects the initial statistics of the adapter weights. Therefore, we reformulate LoRA initialization as a data-aware output-moment calibration problem. We introduce GME-Init, which estimates effective response matrices under coordinated global probes and computes approximate layer-wise scale and asymmetry corrections. Centered Gamma initialization converts these corrections into LoRA parameters without changing the LoRA structure, parameter budget, training budget, or inference cost. We evaluate GME-Init on a GLUE [11] subset with RoBERTa-base [12] and examine its plug-in compatibility with AdaLoRA and DoRA. We also evaluate remote sensing visual question answering and image captioning with Qwen2.5-VL-3B-Instruct [13]. These experiments cover VRSBench-VQA, VRSBench-Caption [14], and UCM-Caption [15] and assess transfer to multimodal domain-specific information understanding.

## 2. Related Work

### 2.1. Parameter-Efficient Adaptation of Large Models

PEFT reduces the memory, computational, and storage costs of adapting large models by training only a small set of newly introduced or reparameterized parameters [1]. Representative approaches include adapters, prefix tuning, prompt tuning, BitFit, ${\left(\mathrm{IA}\right)}^{3}$, and unified or hybrid PEFT frameworks [16,17,18,19,20,21,22,23,24,25,26]. Models such as BERT, RoBERTa, T5, and GPT-style architectures have made efficient transfer to downstream information-processing tasks increasingly important [12,27,28,29]. LoRA represents each weight update as the product of two low-rank matrices, freezes the original weight matrix, and trains only the low-rank branch. LoRA has been widely adopted for natural language understanding, generation, multimodal tasks, and practical deployment [2,3].

Existing LoRA variants mainly improve adaptation capacity or resource efficiency through rank allocation, scaling, optimization, weight decomposition, or storage reduction. AdaLoRA dynamically allocates low-rank budgets. DyLoRA, rsLoRA, and QLoRA address dynamic rank selection, rank-dependent scaling, and quantized fine-tuning, respectively [30,31,32,33]. Recent surveys have reviewed LoRA variants in terms of parameter efficiency, rank allocation, optimization, scaling, weight decomposition, and deployment [3,34,35]. Other methods such as LoRA+, VeRA, and DoRA improve optimization dynamics, parameter sharing, or weight parameterization [36,37,38]. However, these developments mainly concern LoRA training or parameterization, and the use of task-specific data to determine the initial statistical state of the low-rank branch remains comparatively less explored.

### 2.2. LoRA Initialization and Data-Aware Initialization

LoRA initialization affects the initial adapter outputs, gradient directions, and subsequent optimization trajectory. Standard LoRA typically initializes *A* from a symmetric random distribution and *B* to zero, producing a zero initial low-rank update. Recent methods based on weight structure construct more informative initialization subspaces from pretrained parameters. PiSSA places the principal singular components of the pretrained weight matrix into the trainable low-rank branch, whereas OLoRA uses QR decomposition to construct an orthonormal adaptation subspace [4,5]. These methods exploit the geometric structure of pretrained weights, and do not require downstream calibration samples; however, their initialization is primarily determined by weight-space information and does not explicitly adapt the output statistics of target layers to the representation distribution induced by the downstream task.

More recent studies make LoRA initialization task-aware by incorporating downstream activations, covariance information, or optimization signals. EVA constructs initialization subspaces from downstream activation vectors and uses explained variance to identify informative directions [6]. CorDA incorporates input-activation covariance into context-oriented weight decomposition, while ConsNoTrainLoRA derives data-dependent initialization from pretrained weights, downstream activations, and constraint-based formulations [8,9]. LoRA-GA uses downstream gradient information to align the initial low-rank optimization direction with that of full-parameter fine-tuning, whereas LoRA-DA uses target-domain data and task-dependent optimization statistics to construct a data-aware initialization [7,10]. These approaches demonstrate that downstream information can improve LoRA initialization beyond random or purely weight-based schemes.

Existing data-aware methods mainly operate within input-activation, covariance-weight decomposition, gradient, or parameter spaces. They aim to identify informative low-rank directions, preserve task-relevant weight components, or improve initial gradient alignment. However, they do not formulate LoRA initialization as the joint calibration of layer-wise output variance and skewness. GME-Init instead estimates the second- and third-order statistics of target-layer outputs and converts the required corrections into layer-specific initialization scale and asymmetry. In this way, it complements weight-, activation-, covariance-, and gradient-based strategies by operating directly in output-moment space. The key distinction is the object being calibrated, not merely the downstream statistic being used. Existing methods construct, preserve, or align low-rank directions, whereas GME-Init calibrates the observable statistical effect of the initialized adapters in output space.

### 2.3. Statistical Moments and Data-Aware Initialization

More generally, studies on neural network initialization have shown that the initial weight scale, variance propagation, and layer-output statistics affect optimization stability and trainability. Xavier initialization, He initialization, and orthogonal initialization all determine weight statistics from architectural assumptions, while LSUV additionally uses data-dependent activation measurements to calibrate layer-wise variance [39,40,41,42]. While these studies establish the importance of controlling the statistical state of a model before training, conventional initialization methods primarily target full-network signal propagation and usually emphasize variance or approximate dynamical stability; thus, they are not specifically designed for low-rank adapter branches or task-dependent asymmetry in target-layer representations. GME-Init extends the data-aware calibration principle to LoRA initialization and jointly considers variance and skewness, allowing the initial low-rank weights to reflect both the scale and asymmetry of task-specific output distributions.

### 2.4. Text and Multimodal Information Understanding

GLUE is a widely used multitask benchmark for natural language understanding. It covers inference, semantic similarity, linguistic acceptability, and sentiment classification, and can reflect model transfer ability across different text information-processing scenarios [11]. RoBERTa-base is a well-optimized pretrained language model and is commonly used to evaluate PEFT methods on text understanding tasks [12]. Beyond text tasks, multimodal information understanding has become an important application direction for large models.

In addition to language model adaptation, transfer learning and multimodal fusion have been applied to heterogeneous information processing scenarios. Explainable deep reinforcement learning has been combined with transfer learning for climate forecasting from satellite and geospatial data. Quantum-enhanced multimodal fusion has also been explored for fake news detection using textual, visual, and acoustic information [43,44]. Although these studies address different downstream applications rather than PEFT initialization, they demonstrate the broader importance of adapting learned representations to heterogeneous task data.

Remote sensing vision–language models jointly model the spatial textures, ground objects, scene semantics, and natural language instructions or descriptions of remote sensing images. GeoChat, RS-LLaVA, EarthGPT, and LHRS-Bot show that vision–language modeling is becoming an important direction for intelligent remote sensing interpretation [45,46,47,48].

Several remote sensing VLMs, including GeoChat, RS-LLaVA, and LHRS-Bot, use LoRA-based fine-tuning in order to efficiently adapt pretrained multimodal models to remote sensing data [45,46,48]. These studies demonstrate the practical value of LoRA for domain-specific multimodal adaptation; however, they mainly focus on architecture design, instruction tuning, dataset construction, and downstream performance rather than low-rank branch initialization. In contrast, GME-Init studies how task-induced output statistics can determine the initial scale and asymmetry of LoRA parameters before fine-tuning.

This paper uses both GLUE and remote sensing vision–language tasks to evaluate GME-Init from the perspectives of text information processing and multimodal domain-specific information understanding.

## 3. Method

**Figure 1:**
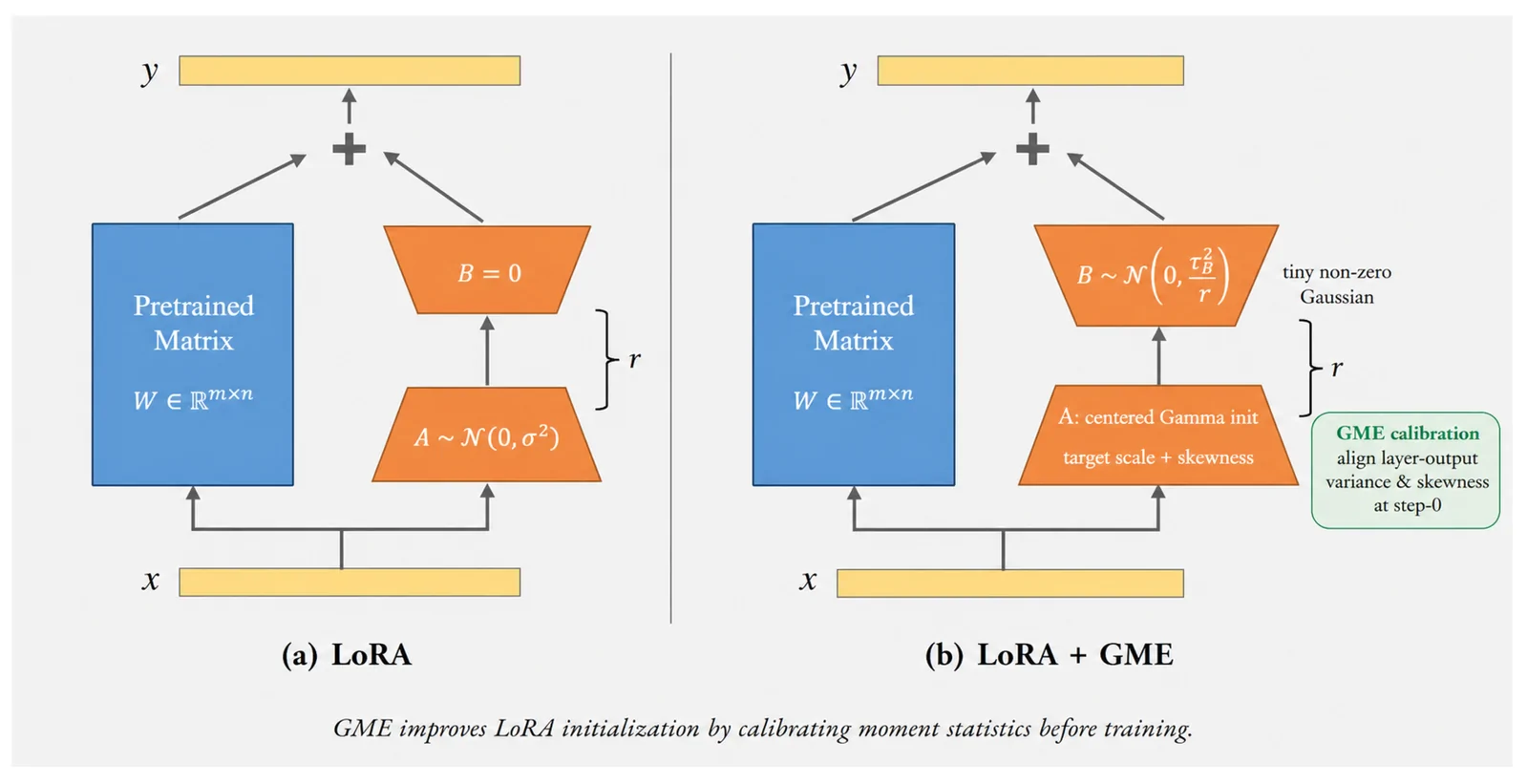
Schematic comparison between standard LoRA initialization and GME-Init. Standard LoRA initializes *A* with a symmetric distribution, such as Gaussian or Kaiming initialization, and initializes *B* to zero, resulting in a zero initial low-rank update. GME-Init initializes *A* with a centered Gamma distribution and initializes *B* with a tiny non-zero Gaussian distribution, where layer-wise initialization statistics are determined by calibrating the output variance and skewness of target layers.

This paper proposes Gamma-Moment Equalization Initialization (GME-Init), a layer-output statistics calibration method for LoRA-style PEFT. Standard LoRA samples *A* from a symmetric distribution, such as a Gaussian or Kaiming distribution, and sets *B* to zero; thus, the initial low-rank update satisfies ΔW=0 [2]. This setting avoids disrupting pretrained representations, but does not reflect the influence of the input feature distribution on initialization. After inputs pass through the pretrained model and its nonlinear transformations, target-layer outputs may exhibit task-dependent variance and skewness. GME-Init treats LoRA initialization as a reduced-order calibration problem in output-moment space, estimates the variance and skewness of each target layer, measures effective layer-wise responses under coordinated global probes, and computes approximate scale and asymmetry corrections. Figure 1 compares standard LoRA initialization with GME-Init.

### 3.1. Overall Framework and Notation

This subsection defines the layer-output statistics calibrated by GME-Init. Let L denote the target LoRA-adapted layers within a moment domain. For encoder-only models, L may contain the encoder layers equipped with LoRA. For decoder-only models, it may contain the corresponding decoder layers. Encoder–decoder models usually have different representation distributions on the two sides. Therefore, we treat the encoder and decoder as separate moment domains and calibrate them independently.

For a calibration mini-batch, we denote the output tensor of the *l*-th target LoRA-adapted layer as $H_{l}\in R^{B\times T\times d_{l}}$, where *B*, *T*, and $d_{l}$ are the batch size, sequence length, and hidden dimension, respectively. For text inputs, padding positions are excluded using the attention mask. For vision–language inputs, the same rule is applied to valid language tokens after multimodal preprocessing. Unless otherwise specified, the empirical moments are computed by flattening all valid token–hidden entries from all calibration batches for the same target layer. Let $\Omega_{l}$ denote this set of valid scalar entries and let $N_{l}=\left|\Omega_{l}\right|$. The empirical mean is

(1) $${\hat{\mu }}_{l}=\frac{1}{N_{l}}\sum_{i\in \Omega_{l}}H_{l,i}.$$

We focus on the second- and third-order statistics of the layer output and define the variance $v_{l}$ and skewness $g_{l}$ as

(2) $$v_{l}=\frac{1}{N_{l}}\sum_{i\in \Omega_{l}}{\left(H_{l,i}-{\hat{\mu }}_{l}\right)}^{2},$$

(3) $$g_{l}=\frac{\frac{1}{N_{l}}\sum_{i\in \Omega_{l}}{\left(H_{l,i}-{\hat{\mu }}_{l}\right)}^{3}}{{\left(v_{l}+\epsilon \right)}^{3/2}},$$

where ϵ is a numerical stability term. Skewness characterizes the asymmetry of the output distribution: $g_{l}>0$ indicates right skew, $g_{l}<0$ indicates left skew, and $g_{l}\approx 0$ indicates an approximately symmetric distribution.

Before initialization, we draw a small calibration set from the training split and perform forward passes without gradients to obtain the baseline statistics $v_{l}^{\left(0\right)}$ and $g_{l}^{\left(0\right)}$ for each target layer. These statistics already include the joint influence of the pretrained backbone, input data, residual connections, normalization layers, and activation functions along with the current LoRA injection position. Therefore, the subsequent response modeling does not separately decompose the statistical contribution of the backbone itself. Within the same moment domain, we use layer-wise medians to define robust common targets:

(4) $$v^{*}={\mathrm{median}}_{l\in L}\left(v_{l}^{\left(0\right)}\right),\;g^{*}={\mathrm{median}}_{l\in L}\left(g_{l}^{\left(0\right)}\right).$$

The median operation is performed across target LoRA-adapted layers or modules within the same moment domain, rather than across tokens or hidden dimensions. Thus, token–hidden entries are used to estimate the moment of each individual layer, while the layer-wise median defines the shared target moment within a domain. GME-Init aims to move the target-layer output statistics toward $v^{*}$ and $g^{*}$ at initialization. This reduces the layer-wise output-moment inconsistency and better aligns the scale and asymmetry of the initial LoRA weights with the representation distribution of the current task.

### 3.2. Effective Response Matrix Estimation Under Coordinated Probes

We next operationalize the layer-wise moment targets by estimating how each target layer responds to controlled changes in the LoRA initialization scale and asymmetry. Let $\left({\bar{\sigma }}^{2},\bar{\gamma }\right)$ denote the two scalar controls of a coordinated probe applied simultaneously to all target adapters in L. Each target adapter receives the same probe scale and asymmetry, but we record the resulting output moments separately at every layer. Because an explicit analytical model of the complete network response is intractable, we express the variance and skewness at layer *l* as functions of the coordinated probe controls:

(5) $$v_{l}={\tilde{F}}_{l}\left({\bar{\sigma }}^{2},\bar{\gamma }\right),\;g_{l}={\tilde{G}}_{l}\left({\bar{\sigma }}^{2},\bar{\gamma }\right).$$

Applying a first-order bivariate Taylor expansion around the baseline point $\left({\bar{\sigma }}^{2},\bar{\gamma }\right)=\left(0,0\right)$ gives

(6) $$v_{l}-v_{l}^{\left(0\right)}\approx a_{l}{\bar{\sigma }}^{2}+b_{l}\bar{\gamma },$$

(7) $$g_{l}-g_{l}^{\left(0\right)}\approx c_{l}{\bar{\sigma }}^{2}+d_{l}\bar{\gamma }.$$

Thus, the change in output moments can be written in the following effective response-matrix form:

(8) $$\left[\begin{array}{c} \Delta v_{l}\\ \Delta g_{l} \end{array}\right]\approx \underset{M_{l}}{\underset{︸}{\left[\begin{array}{cc} a_{l} & b_{l}\\ c_{l} & d_{l} \end{array}\right]}}\left[\begin{array}{c} {\bar{\sigma }}^{2}\\ \bar{\gamma } \end{array}\right].$$

The coefficients $a_{l}$ and $c_{l}$ describe the effective variance and skewness responses at layer *l* to the coordinated scale probe. The coefficients $b_{l}$ and $d_{l}$ describe the corresponding responses to the coordinated asymmetry probe. Because all target adapters are probed simultaneously, $M_{l}$ is not the isolated causal Jacobian of the *l*-th adapter. It instead represents the effective local response of the moments observed at layer *l* along a coordinated global direction. At downstream layers, this response also includes effects propagated from upstream adapter perturbations. Therefore, GME uses $M_{l}$ as an effective layer-wise approximation, not as an exact derivative with respect to a single adapter.

GME retains both main-response and cross-response terms in $M_{l}$. To examine the utility of the cross-response terms, we also construct a low-order approximation, denoted GME-LO. GME-LO retains only the effective main-response paths from the coordinated scale probe to output variance and from the coordinated asymmetry probe to output skewness, while ignoring the cross terms corresponding to $b_{l}$ and $c_{l}$.

The remaining task is to estimate the coefficients of this reduced two-parameter model. We use a small number of coordinated gradient-free forward probes. Baseline and probe moments are computed from the same calibration data with the same aggregation rule, so their differences capture the total local response of each observed layer. This response includes the direct contribution of the adapter at that layer, and for downstream layers includes the propagated contributions from upstream adapters. We first run a baseline forward pass to obtain $v_{l}^{\left(0\right)}$ and $g_{l}^{\left(0\right)}$, then apply a coordinated scale-only probe by assigning $\left(\bar{\sigma },\bar{\gamma }\right)=\left(\sigma_{\mathrm{probe}},0\right)$ to every target adapter. The probe yields $v_{l}^{\left(\sigma \right)}$ and $g_{l}^{\left(\sigma \right)}$ at each layer, from which we estimate

(9) $$a_{l}=\frac{v_{l}^{\left(\sigma \right)}-v_{l}^{\left(0\right)}}{\sigma_{\mathrm{probe}}^{2}+\epsilon },\;c_{l}=\frac{g_{l}^{\left(\sigma \right)}-g_{l}^{\left(0\right)}}{\sigma_{\mathrm{probe}}^{2}+\epsilon }.$$

A coordinated scale-plus-asymmetry probe next assigns $\left(\bar{\sigma },\bar{\gamma }\right)=\left(\sigma_{\mathrm{probe}},\gamma_{\mathrm{probe}}\right)$ to all target adapters and records $v_{l}^{\left(\sigma ,\gamma \right)}$ and $g_{l}^{\left(\sigma ,\gamma \right)}$. To estimate the marginal effective response along the asymmetry direction, the change already produced by the scale-only probe is subtracted:

(10) $$b_{l}=\frac{v_{l}^{\left(\sigma ,\gamma \right)}-v_{l}^{\left(\sigma \right)}}{\gamma_{\mathrm{probe}}+\epsilon },\;d_{l}=\frac{g_{l}^{\left(\sigma ,\gamma \right)}-g_{l}^{\left(\sigma \right)}}{\gamma_{\mathrm{probe}}+\epsilon }.$$

Let

(11) $$M_{l}=\left[\begin{array}{cc} a_{l} & b_{l}\\ c_{l} & d_{l} \end{array}\right],\;y_{l}=\left[\begin{array}{c} v^{*}-v_{l}^{\left(0\right)}\\ g^{*}-g_{l}^{\left(0\right)} \end{array}\right].$$

Based on the effective response matrix, GME computes an approximate layer-wise initialization correction toward the common target moments:

(12) $$\left[\begin{array}{c} \sigma_{w,l}^{2}\\ \gamma_{w,l} \end{array}\right]≜M_{l}^{-1}y_{l}.$$

This solution is a layer-wise surrogate correction derived from the effective response measured at layer *l*, rather than the exact inversion of the full cross-layer response Jacobian. The approximation assumes that, within the small-probe regime, the effective response observed at each layer provides a useful local direction for reducing its moment discrepancy. The influence of residual higher-order and cross-layer interactions is limited through the tiny non-zero scale of *B*, parameter clipping, numerical damping, and the optional moment refinement step.

When $\det\left(M_{l}\right)=a_{l}d_{l}-b_{l}c_{l}$ is not close to zero, the surrogate correction is computed explicitly as

(13) $$\sigma_{w,l}^{2}=\frac{d_{l}\left(v^{*}-v_{l}^{\left(0\right)}\right)-b_{l}\left(g^{*}-g_{l}^{\left(0\right)}\right)}{a_{l}d_{l}-b_{l}c_{l}},$$

(14) $$\gamma_{w,l}=\frac{-c_{l}\left(v^{*}-v_{l}^{\left(0\right)}\right)+a_{l}\left(g^{*}-g_{l}^{\left(0\right)}\right)}{a_{l}d_{l}-b_{l}c_{l}}.$$

Non-negativity and clipping are then applied:

(15) $$\sigma_{w,l}\leftarrow \mathrm{clip}\left(\sqrt{\max(\sigma_{w,l}^{2},0)},\sigma_{\min},\sigma_{\max}\right),$$

(16) $$\gamma_{w,l}\leftarrow \mathrm{clip}\left(\gamma_{w,l},-\gamma_{\max},\gamma_{\max}\right).$$

For numerical stability, we treat an effective response matrix as ill-conditioned when $|\det(M_{l})|<\delta$ or when its condition number exceeds a preset threshold. We first apply a damped inverse. If the resulting scale or asymmetry parameter lies outside the valid range, we use the diagonal GME-LO approximation instead. Finally, we clip $\sigma_{w,l}$ and $\gamma_{w,l}$ to predefined ranges in order to prevent an approximately singular response matrix from producing unstable initialization parameters.

The effective response matrix is intended to provide a local first-order approximation along the coordinated probe directions rather than an exact representation of the full cross-layer nonlinear response. Let

(17) $$z={\left[\begin{array}{cc} {\bar{\sigma }}^{2} & \bar{\gamma } \end{array}\right]}^{⊤}$$

denote the two coordinated probe controls, and define

(18) $$m_{l}^{e}\left(z\right)={\left[\begin{array}{cc} F_{l}^{e}\left(z\right) & G_{l}^{e}\left(z\right) \end{array}\right]}^{⊤}$$

as the output variance and skewness observed at layer *l* under the coordinated probe. Here, $F_{l}^{e}\left(z\right)={\tilde{F}}_{l}\left({\bar{\sigma }}^{2},\bar{\gamma }\right)$ and $G_{l}^{e}\left(z\right)={\tilde{G}}_{l}\left({\bar{\sigma }}^{2},\bar{\gamma }\right)$. Let $J_{l}^{e}=\nabla_{z}m_{l}^{e}\left(0\right)$ denote the exact effective Jacobian with respect to the two probe controls. We write its finite-probe estimate as $M_{l}=J_{l}^{e}+E_{l}$, where $E_{l}$ is the response estimation error. If $\nabla_{z}m_{l}^{e}\left(z\right)$ is locally $L_{l}$-Lipschitz continuous around 0, Taylor’s theorem gives

(19) $${∥m_{l}^{e}\left(z\right)-m_{l}^{e}\left(0\right)-M_{l}z∥}_{2}\leq \frac{L_{l}}{2}{∥z∥}_{2}^{2}+{∥E_{l}∥}_{2}{∥z∥}_{2}.$$

Therefore, the approximation error along the coordinated probe subspace contains a second-order local remainder and a finite-probe estimation term. Small probe magnitudes keep the approximation within the local regime and support the use of $M_{l}$ as an effective local response. In this way, the initialization scale and asymmetry are obtained from the measured output-moment calibration equations rather than from a fixed heuristic. The residual reflects higher-order local effects and finite-probe estimation errors. Because the layer-wise solution is a surrogate correction rather than an exact inversion of the full cross-layer response, damping and clipping keep it within a bounded local regime.

The conditioning of $M_{l}$ determines how strongly the moment estimation error and finite-probe error may be amplified when the surrogate correction is computed. For the damped inverse

(20) $$D_{\lambda ,l}={\left(M_{l}^{⊤}M_{l}+\lambda I\right)}^{-1}M_{l}^{⊤},\;\lambda >0,$$

its spectral norm satisfies

(21) $${∥D_{\lambda ,l}∥}_{2}=\max_{i}\frac{s_{i}}{s_{i}^{2}+\lambda }\leq \frac{1}{2\sqrt{\lambda }},$$

where $s_{i}$ denotes a singular value of $M_{l}$. Therefore, damping bounds the amplification of perturbations when the effective response matrix contains a small singular value. Clipping $\sigma_{w,l}$ and $\gamma_{w,l}$ to predefined intervals further bounds the magnitude of the layer-wise surrogate correction. If the effective response remains unreliable after damping, the GME-LO fallback removes the cross-response terms and applies the more conservative diagonal approximation. Together, damping, clipping, and fallback prevent a poorly conditioned effective response estimate from producing unbounded initialization parameters.

GME-Init is primarily a one-step initialization procedure, not an iterative optimization algorithm. We do not claim global convergence for the complete nonlinear cross-layer system. When enabled, the optional moment-refining procedure can be interpreted as a projected fixed-point iteration on the reduced-order response model. If the local Jacobian of the refinement map has spectral norm below one within the bounded feasible region, the update is locally contractive up to the higher-order approximation and finite-probe estimation errors. Projection onto the predefined scale–asymmetry intervals is non-expansive, so clipping does not increase the distance to a feasible local fixed point. This interpretation provides a local convergence condition for refinement without implying global convergence of the full nonlinear model.

### 3.3. Centered Gamma Initialization

After obtaining bounded layer-wise scale and asymmetry targets, we translate them into probability distributions for the LoRA matrices. The sampling scheme must control variance and asymmetry while keeping the mean close to zero; therefore, we draw *A* from a centered Gamma distribution and *B* from a tiny non-zero Gaussian distribution. This step converts the calibrated statistical targets into concrete data-aware asymmetric LoRA parameters. For a pretrained weight matrix $W_{l}\in R^{d_{\mathrm{out}}\times d_{\mathrm{in}}}$, LoRA uses two low-rank matrices, $A_{l}\in R^{r\times d_{\mathrm{in}}}$ and $B_{l}\in R^{d_{\mathrm{out}}\times r}$:

(22) $$\Delta W_{l}=s_{l}B_{l}A_{l}$$

where *r* is the LoRA rank and $s_{l}$ is the LoRA scaling factor, which can follow the conventional α/r or be set as a layer-wise scaling coefficient.

For $A_{l}$, we use centered Gamma initialization. The skewness of a Gamma distribution is $2/\sqrt{k_{l}}$, so the shape parameter can be inferred from the target skewness magnitude:

(23) $$k_{l}={\left(\frac{2}{\max(|\gamma_{w,l}|,\gamma_{\min})}\right)}^{2}.$$

To make the standard deviation of $A_{l}$ close to $\sigma_{w,l}$, the scale parameter is set as

(24) $$\theta_{l}=\frac{\sigma_{w,l}}{\sqrt{k_{l}}}.$$

We then sample from the Gamma distribution,

(25) $$X_{l}∼\Gamma \left(k_{l},\theta_{l}\right),$$

and apply centering and direction control:

(26) $$A_{l}={\mathrm{sgn}}_{0}\left(\gamma_{w,l}\right)\left(X_{l}-k_{l}\theta_{l}\right)$$

where ${\mathrm{sgn}}_{0}\left(\gamma \right)=1$ if γ≥0 and ${\mathrm{sgn}}_{0}\left(\gamma \right)=-1$ otherwise. This construction gives $A_{l}$ an approximately zero mean, a standard deviation close to $\sigma_{w,l}$, and a skewness direction controlled by $\gamma_{w,l}$.

If $B_{l}$ remains exactly zero, then $\Delta W_{l}=s_{l}B_{l}A_{l}=0$, and output-moment calibration cannot affect layer outputs at initialization. Therefore, we use a tiny non-zero Gaussian initialization

(27) $$B_{l,ij}∼N\left(0,\frac{\tau_{B}^{2}}{r}\right),$$

where $\tau_{B}$ is a small constant. This design produces a non-zero LoRA perturbation before training, which allows initialization to adjust the output moments. The small value of $\tau_{B}$ limits $∥\Delta W_{l}∥$ and avoids substantially disturbing the pretrained representation.

Algorithm 1 summarizes the full GME-Init procedure. The algorithm relies only on calibration forward passes and does not require label-based backpropagation. The final output remains standard LoRA matrices $A_{l}$ and $B_{l}$, so the subsequent training and inference interfaces are unchanged and no additional trainable parameters are introduced.

### 3.4. Application to Different Information-Processing Tasks

GME-Init depends only on target-layer output-moment estimation, meaning that it can be applied to LoRA modules in text and multimodal vision–language models. In the experiments on text understanding, we apply it to the transformer attention projections of RoBERTa-base. We draw calibration samples from the task training split and process them in gradient-free forward passes. These passes collect the variance and skewness of the target-layer outputs without label-based backpropagation or additional training epochs.

In the remote sensing vision–language experiments, we apply GME-Init to LoRA modules in the language backbone of Qwen2.5-VL-3B-Instruct. The visual encoder is frozen by default and excluded from the LoRA target modules. This choice separates the effect of initialization from changes caused by visual tower fine-tuning. We format VQA samples as “image + question → short answer” instructions and captioning samples as “image + remote sensing captioning instruction → image description” instructions. By default, LoRA targets the language backbone’s attention and MLP projections: q_proj, k_proj, v_proj, o_proj, gate_proj, up_proj, and down_proj. All compared PEFT methods share the same base model, target modules, rank, scaling, dropout, and training budget, differing only in the initialization strategy or PEFT variant.
**Algorithm 1** GME-Init with explicit A/B initialization**Require:** Model with LoRA adapters ${\left\{\left(A_{l},B_{l}\right)\right\}}_{l\in L}$, calibration data $D_{cal}$, probe scale $\sigma_{p}$, probe skewness $\gamma_{p}$, tiny Gaussian scale $\tau_{B}$
**Ensure:** Initialized adapters ${\left\{\left(A_{l},B_{l}\right)\right\}}_{l\in L}$
  1:Collect baseline output moments $\left(v_{l}^{0},g_{l}^{0}\right)$ over all valid token–hidden entries on $D_{cal}$; set $v^{*}\leftarrow {\mathrm{median}}_{l}\left(\left\{v_{l}^{0}\right\}\right)$, $g^{*}\leftarrow {\mathrm{median}}_{l}\left(\left\{g_{l}^{0}\right\}\right)$  2:**Coordinated Probe-A:** simultaneously assign $A_{l}∼\mathrm{CenteredGamma}\left(\sigma_{p},0\right)$ and $B_{l}∼N\left(0,{\left(\tau_{B}/\sqrt{r_{l}}\right)}^{2}\right)$ to all target adapters; collect the layer-wise moments $\left(v_{l}^{A},g_{l}^{A}\right)$  3:**Coordinated Probe-B:** simultaneously assign $A_{l}∼\mathrm{CenteredGamma}\left(\sigma_{p},\gamma_{p}\right)$ and $B_{l}∼N\left(0,{\left(\tau_{B}/\sqrt{r_{l}}\right)}^{2}\right)$ to all target adapters; collect the layer-wise moments $\left(v_{l}^{B},g_{l}^{B}\right)$  4:**for** l∈L **do**  5:     Estimate the effective layer-wise response matrix $M_{l}=\left[\begin{array}{cc} a_{l} & b_{l}\\ c_{l} & d_{l} \end{array}\right]$ from the coordinated probe differences  6:     Compute the approximate correction ${\left[\sigma_{l}^{2},\gamma_{l}\right]}^{⊤}≜M_{l}^{-1}{\left[v^{*}-v_{l}^{0},\;g^{*}-g_{l}^{0}\right]}^{⊤}$; use a damped inverse or diagonal fallback if $M_{l}$ is ill-conditioned  7:     Clip $\sigma_{l}$ and $\gamma_{l}$ to predefined valid ranges; set calibration ratio $\rho_{l}\leftarrow \sigma_{l}/\sigma_{p}$  8:     **Final init:** sample $A_{l}∼\mathrm{CenteredGamma}\left(\sigma_{l},\gamma_{l}\right)$ and $B_{l}∼N\left(0,{\left(\tau_{B}\rho_{l}/\sqrt{r_{l}}\right)}^{2}\right)$  9:**end for**10:**if** moment refinement is enabled **then**11:     Repeat a few output-moment refinement steps on $\sigma_{l},\gamma_{l}$ toward $\left(v^{*},g^{*}\right)$12:**end if**13:**return** initialized adapters ${\left\{\left(A_{l},B_{l}\right)\right\}}_{l\in L}$


## 4. Experiments

This section evaluates GME-Init from four perspectives. We compare it with standard LoRA, weight-space initialization methods, and related data-aware methods on GLUE text understanding tasks. We additionally examine its plug-in compatibility with AdaLoRA and DoRA. A component ablation then separates the effects of non-zero *B* initialization, asymmetric *A* sampling, moment calibration, and effective response-matrix modeling. Remote sensing VQA and image captioning experiments assess transfer to multimodal domain-specific information understanding. Finally, we measure output-moment mismatch before and after GME-Init to verify the intended initialization-time correction.

### 4.1. Experimental Setup

For text understanding, we use RoBERTa-base [12] as the backbone and evaluate it on a GLUE [11] subset containing RTE, MRPC, CoLA, SST-2, and STS-B. We report accuracy for RTE and SST-2, F1 for MRPC, Matthews correlation for CoLA, and Pearson correlation for STS-B.

For all GLUE tasks, we use the official training split for fine-tuning and report performance on the official development set. Calibration uses only the input sequences and attention masks in gradient-free forward passes, without using any labels, class information, or development-set samples. After initialization, the calibration samples remain part of the full training split used for gradient-based fine-tuning.

Task-wise GLUE results are reported as mean ± standard deviation over five random seeds and converted to a percentage scale, where higher is better. The component ablation reports the corresponding five-task average for compactness. The standard LoRA experiments compare LoRA, PiSSA, OLoRA, GME-LO, and GME with ranks r∈{4,8,16}. All methods inject adapter parameters only into the query, key, value, and attention-output projection layers of the transformer attention module [49] and use the same training budget. To further assess GME-Init against related data-aware LoRA initialization methods, we additionally compare it with ConsNoTrainLoRA, CorDA, and LoRA-GA at rank r=8. Standard LoRA is included as the reference baseline. All methods are evaluated using the same RoBERTa-base backbone, target modules, training budget, evaluation protocol, and five random seeds.

For all experiments involving GME-Init, the probe, initialization, clipping, damping, and refinement hyperparameters are held fixed across the GLUE and remote sensing settings. The default calibration-set size is 512 samples per GLUE task and 128 samples for the remote sensing experiments, except for in the calibration-sample ablation reported in Section 4.8. Specifically, GME-Init uses a probe scale of $\sigma_{p}=0.02$, a probe skewness of $\gamma_{p}=0.2$, and a Gaussian scale of $\tau_{B}=10^{-4}$ for the initialization of *B*. For the final initialization, the standard deviation of $B_{l}$ is set to $\tau_{B}\left(\sigma_{l}/\sigma_{p}\right)/\sqrt{r}$. The estimated response coefficients are clipped to [−10,10], while the solved scale and asymmetry parameters are clipped to $\sigma_{l}\in \left[0.005,0.05\right]$ and $\gamma_{l}\in \left[-0.8,0.8\right]$, respectively. A response matrix is treated as ill-conditioned when $\left|\det\left(M_{l}\right)\right|<10^{-6}$ or $\mathrm{cond}\left(M_{l}\right)>10^{6}$. In such cases, we use damping with $\lambda =10^{-6}$, followed by the GME-LO fallback when necessary. The calibration samples are drawn without replacement from the corresponding training split using the experiment seed, and the calibration forward batch size is 32. Moment refinement uses two steps with $\eta_{v}=\eta_{g}=0.5$.

For the plug-in experiments, we integrate GME-Init into AdaLoRA [30] and DoRA [38]. In each comparison, DefaultInit and GMEInit use the same backbone, task, training hyperparameters, and random seeds. They differ only in initialization. This design tests whether output-moment calibration is limited to standard LoRA or can also support methods based on low-rank budget allocation and weight reparameterization.

For the remote sensing vision–language experiments, we use Qwen2.5-VL-3B-Instruct [13] as the unified backbone. The tasks include VRSBench-VQA, VRSBench-Caption [14], and UCM-Caption [15]. Compared methods include zero-shot, LoRA, LoRA + GME, DoRA, DoRA + GME, and LoRA+PiSSA. To extend the comparison to data-aware initialization strategies in the remote sensing domain, we additionally evaluate ConsNoTrainLoRA and CorDA on VRSBench-VQA and ConsNoTrainLoRA in the 1k and 5k VRSBench-Caption settings [8,9]. Except for the zero-shot method, all remote sensing PEFT methods use the same rank, target modules, training budget, and evaluation protocol. All remote sensing results are reported as mean ± standard deviation over five random seeds. VQA is evaluated with Exact Match, Contains Match, and Token F1. Captioning is evaluated with BLEU-4, ROUGE-L, METEOR-lite, and CIDEr. All remote sensing metrics retain the original scale produced by the evaluation scripts and are not converted to percentages. Higher values indicate better performance.

We also quantify the one-time computational overhead of GME-Init. The measurements use RoBERTa-base with rank-8 LoRA adapters on the query, key, value, and attention-output projections. We used an NVIDIA GeForce RTX 4090 GPU with 24 GB of memory, 512 calibration samples, and a calibration batch size of 32. Results were averaged over five GLUE tasks and five random seeds. Standard LoRA initialization required 0.685 s. GME-Init required 0.152 s for calibration-data preprocessing and 5.339 s for calibration, giving a total of 5.491 s. This duration is approximately eight times that of standard LoRA initialization. The ratio appears large because standard initialization is extremely short; in absolute terms, GME-Init requires only about 5.5 s before fine-tuning. Relative to a three-hour fine-tuning run, this stage accounts for approximately 0.051% of the total runtime, and the proportion is lower for longer runs. Calibration increased peak GPU memory by 91.65 MB, or approximately 0.37% of the nominal GPU capacity, and required only 0.165 MB of additional CPU tensor storage. After calibration, the forward intermediates and moment collection hooks are removed. The initialized LoRA matrices retain the same shapes and parameter count as standard LoRA.

In terms of computational complexity, standard LoRA initialization requires $O(P_{\mathrm{LoRA}})$ operations to initialize the adapter parameters, where $P_{\mathrm{LoRA}}$ denotes the total number of parameters in matrices *A* and *B*. GME-Init additionally performs one baseline calibration sweep, two coordinated probe sweeps, and *J* optional moment-refinement sweeps over the calibration set. Therefore, its initialization-time complexity is $O(\left(3+J\right)C_{\mathrm{fwd}}\left(N_{\mathrm{cal}}\right)+P_{\mathrm{LoRA}})$, where $C_{\mathrm{fwd}}\left(N_{\mathrm{cal}}\right)$ denotes the cost of one gradient-free forward sweep over $N_{\mathrm{cal}}$ calibration samples. The inversion of each 2×2 response matrix has constant cost, giving only O(|L|) additional operations across the target layers. Since all calibration sweeps are gradient-free and processed in mini-batches, GME-Init does not store backward graphs or optimizer states.

Based on these results, GME-Init achieves improved average downstream performance with negligible one-time runtime overhead relative to the multi-hour fine-tuning process, while leaving the trainable parameter count, per-step training computation, and inference-time computation unchanged.

### 4.2. Main Results on GLUE Text Understanding

Table 1 reports the RoBERTa-base results on five GLUE tasks. GME and GME-LO are not new adapter structures; they are alternative initialization strategies for standard LoRA. Apart from initialization, they use the same injection positions, ranks, and training configurations. Here, Avg. is the arithmetic mean of the five task metrics and summarizes cross-task trends.

GME improves the five-task average over standard LoRA at all three ranks: Avg. increases from 81.54 to 82.31 at r=4, from 82.09 to 83.19 at r=8, and from 82.35 to 83.54 at r=16. The corresponding gains are 0.77, 1.10, and 1.19 percentage points. Because the adapter structure, trainable parameter count, injection positions, ranks, and training hyperparameters remain unchanged, these gains reflect the effect of data-aware asymmetric initialization. While GME does not achieve the best score on every task relative to PiSSA and OLoRA, it obtains the highest Avg. at all three ranks and the best result on all five tasks at r=16. GME-LO yields smaller average gains and remains below full GME. This result supports modeling of both the main effects and cross-effects of scale and asymmetry on output variance and skewness. The following subsection compares GME with additional data-aware initialization methods.

### 4.3. Comparison with Related Data-Aware LoRA Initialization Methods

We further compare GME-Init with ConsNoTrainLoRA, CorDA, and LoRA-GA on the GLUE subset. All experiments use RoBERTa-base with rank r=8. Here, ‘data-aware initialization’ refers broadly to methods that use downstream activation statistics, covariance information, or gradient signals. Standard LoRA serves as the reference baseline. We report mean ± standard deviation over five random seeds.

Table 2 shows that the related data-aware methods exhibit different task-specific strengths: ConsNoTrainLoRA obtains the highest STS-B score, CorDA performs best on SST-2, and LoRA-GA achieves the highest MRPC score. GME obtains the best results on RTE, while CoLA and achieves the highest five-task average of 83.19. This exceeds standard LoRA, ConsNoTrainLoRA, CorDA, and LoRA-GA by 1.10, 0.80, 0.76, and 1.40 percentage points, respectively. Under the matched rank, backbone, target modules, training budget, and evaluation protocol, these results indicate that direct calibration in the output-moment space provides a balanced initialization across the evaluated text understanding tasks.

### 4.4. Plug-In Experiments with AdaLoRA and DoRA

Table 3 presents the results of integrating GME-Init into AdaLoRA and DoRA. DefaultInit denotes the default initialization of the original method, while GMEInit denotes replacing only the initialization with GME-Init.

Under the same training configuration, DoRA-GMEInit outperforms DoRA-DefaultInit on all five tasks and raises Avg. from 81.92 to 82.33. AdaLoRA-GMEInit raises Avg. from 83.26 to 83.99. Its gains on RTE and CoLA are 1.08 and 1.73 percentage points, respectively. Because only the initialization strategy changes in each comparison, the results show that GME-Init is compatible with both low-rank budget allocation and weight reparameterization methods.

### 4.5. Component Ablation and Numerical Stability of GME-Init

Table 4 reports a component ablation on the GLUE subset with RoBERTa-base at rank r=8. The comparison starts from standard LoRA and progressively introduces the main design elements of GME-Init. Using only a tiny non-zero initialization for *B* improves the GLUE average from 82.09 to 82.55, indicating that allowing the low-rank branch to produce a controlled initial perturbation is beneficial. Replacing the symmetric initialization of *A* with a centered Gamma distribution alone obtains an average of 82.51, suggesting that asymmetric sampling by itself is not sufficient to explain the full gain.

The calibration variants further show that moment matching is the main source of improvement. Variance-only calibration and skewness-only calibration reach 82.84 and 82.79, respectively, both exceeding the non-calibrated initialization variants. Full GME obtains the highest average of 83.19, indicating that jointly calibrating variance and skewness with the full effective response matrix is more effective than using either moment alone or a diagonal approximation. This supports the use of a coupled effective-response approximation in output-moment space rather than treating scale and asymmetry as independent initialization heuristics.

To complement the local stability analysis in Section 3.2, we also recorded the activation frequency of the numerical safeguards used in estimating the effective response matrix. Across the reported GLUE and remote sensing settings, ill-conditioned effective response matrices and fallback operations each occurred in fewer than 5% of the target LoRA-adapted layers or modules. The clipping rate for the scale parameter $\sigma_{w,l}$ also remained below 5%, while the clipping rate for the asymmetry parameter $\gamma_{w,l}$ remained below 15%. These results indicate that the direct or damped surrogate response solution is applicable to the large majority of target modules. Fallback is required only for a small number of poorly conditioned effective response matrices, while clipping is activated only occasionally to bound extreme scale or asymmetry corrections. This empirical behavior is consistent with the bounded local correction analysis given in Section 3.2.

### 4.6. Main Results on Remote Sensing VQA

Table 5 reports the main VRSBench-VQA results. Task-specific LoRA adaptation substantially outperforms zero-shot Qwen2.5-VL-3B-Instruct, showing the value of dataset-specific PEFT for remote sensing short-answer VQA. Under matched target modules, rank, and training budget, GME raises Token F1 from 0.5668 to 0.5747, Exact Match from 0.5550 to 0.5605, and Contains Match from 0.5975 to 0.6030. Higher results for Exact Match indicate that more generated answers exactly match the references, while higher Contains Match shows that the answers more often include the correct object category, scene type, or count and higher Token F1 indicates better word-level overlap. Their simultaneous improvement supports the positive effect of GME initialization on short-answer semantic matching across scene recognition, object counting, and ground–object relation questions. Adding GME to DoRA also raises Token F1 from 0.5668 to 0.5696, supporting its use with an existing PEFT variant. Among the additional data-aware baselines, ConsNoTrainLoRA obtains mean Exact Match, Contains Match, and Token F1 scores of 0.5575, 0.5995, and 0.5706, respectively. CorDA obtains 0.4385, 0.4975, and 0.4598. LoRA+GME achieves the highest mean on all three metrics and exceeds ConsNoTrainLoRA by 0.0030, 0.0035, and 0.0041, respectively. This comparison extends the evidence for output-moment calibration from text understanding to remote sensing VQA.

### 4.7. Main Results on Remote Sensing Image Captioning

Table 6 reports VRSBench-Caption results with 1k and 5k training samples. In the 1k low-resource setting, GME raises the CIDEr score of standard LoRA from 0.1743 to 0.1797 and also improves ROUGE-L and METEOR-lite. CIDEr measures n-gram consistency with reference captions, whereas ROUGE-L reflects longer-sequence overlap. METEOR-lite provides a complementary measure of word-level matching. The joint improvements indicate that GME produces captions that better match the scene semantics in the references rather than merely changing generation length. In the 1k setting, ConsNoTrainLoRA obtains a CIDEr score of 0.1764 and the highest METEOR-lite score of 0.4760. LoRA+GME records higher mean BLEU-4, ROUGE-L, and CIDEr scores. PiSSA achieves a slightly higher CIDEr score than LoRA+GME, showing that weight-decomposition initialization remains competitive for this low-resource generation setting. With 5k training samples, LoRA+GME achieves the highest CIDEr score and improves standard LoRA from 0.1861 to 0.1959. It also outperforms LoRA+PiSSA (0.1872) and ConsNoTrainLoRA (0.1830). ConsNoTrainLoRA obtains a slightly higher METEOR-lite mean than LoRA+GME at this scale (0.4829 versus 0.4824), whereas LoRA+GME remains higher in BLEU-4, ROUGE-L, and CIDEr. These results support the effectiveness of output-moment calibration in direct comparisons with another data-aware initialization method for remote sensing captioning.

Table 7 reports cross-dataset captioning results on UCM-Caption. GME improves standard LoRA on all four metrics and raises CIDEr from 3.4732 to 3.5667. This result indicates better caption matching under a different captioning distribution. PiSSA performs slightly better than LoRA+GME on this dataset, showing that weight-decomposition initialization remains competitive for some image captioning distributions. Therefore, we do not claim that GME dominates every strong initialization baseline; instead, we emphasize its consistent gains over standard LoRA under an unchanged structure and training budget together with its competitive and complementary relationship with methods such as PiSSA.

### 4.8. Target Module and Calibration Sample Ablations

Table 8 presents the target module ablation on VRSBench-VQA. GME improves the corresponding LoRA result under every tested configuration. The “Full target set” includes q_proj, k_proj, v_proj, o_proj, gate_proj, up_proj, and down_proj. It achieves the highest absolute Token F1, and is used by default in the main remote sensing experiments. The “Attention q/k/v/o” and “Attention q/v” configurations yield lower absolute performance, but GME still improves their LoRA baselines. Thus, output-moment-aware initialization does not depend on a single target-module configuration.

Table 9 reports the calibration sample ablation on VRSBench-Caption. The CIDEr scores of GME with 32, 128, and 512 calibration samples are 0.1946, 0.1959, and 0.1916, respectively. The 128-sample setting achieves the highest CIDEr, while the 32- and 512-sample settings obtain the highest METEOR-lite and ROUGE-L scores, respectively. The differences across the tested settings remain small, suggesting that the method is not highly sensitive to the number of calibration samples. We use 128 calibration samples by default because it provides the strongest CIDEr result without requiring a large calibration set.

### 4.9. Output-Moment Alignment Analysis

To test whether GME-Init performs the intended output-moment correction, we compare the layer-wise variance and skewness mismatches before and after initialization. For each task or training setting, we first compute the absolute distance between each target layer’s output moment and the corresponding common target, then average these distances across all target layers. The “before” values use the baseline initialization, while the “after” values are measured immediately after GME-Init and before gradient-based fine-tuning. Table 10 reports both mismatches and their relative reductions.

GME-Init reduces both mismatch measures in every reported task and setting. Across the five GLUE tasks, the variance mismatch decreases by 90.71%–99.21%, while the skewness mismatch decreases by 45.62%–89.39%. In the remote sensing settings, the corresponding reduction ranges are 89.54%–98.13% for variance and 62.65%–87.13% for skewness. These consistent reductions show that the calibrated scale and asymmetry move target-layer outputs toward their common moment targets before any gradient update. Variance is corrected particularly strongly, while skewness also decreases substantially despite being a higher-order statistic.

The alignment results provide mechanism-level evidence that complements the downstream results. On GLUE, replacing standard LoRA initialization with GME raises the five-task average by 0.77, 1.10, and 1.19 percentage points at ranks 4, 8, and 16, respectively (Table 1). On VRSBench-VQA, Token F1 rises from 0.5668 to 0.5747 (Table 5). On VRSBench-Caption, CIDEr rises from 0.1743 to 0.1797 with 1k training samples and from 0.1861 to 0.1959 with 5k samples (Table 6). UCM-Caption CIDEr also rises from 3.4732 to 3.5667 (Table 7). Together, the immediate reduction in output-moment mismatch and the subsequent gains under matched training configurations support the intended role of GME-Init. It provides a task-adapted initialization state that improves downstream adaptation in the evaluated settings.

### 4.10. Qualitative Remote Sensing Image Analysis

**Figure 2:**
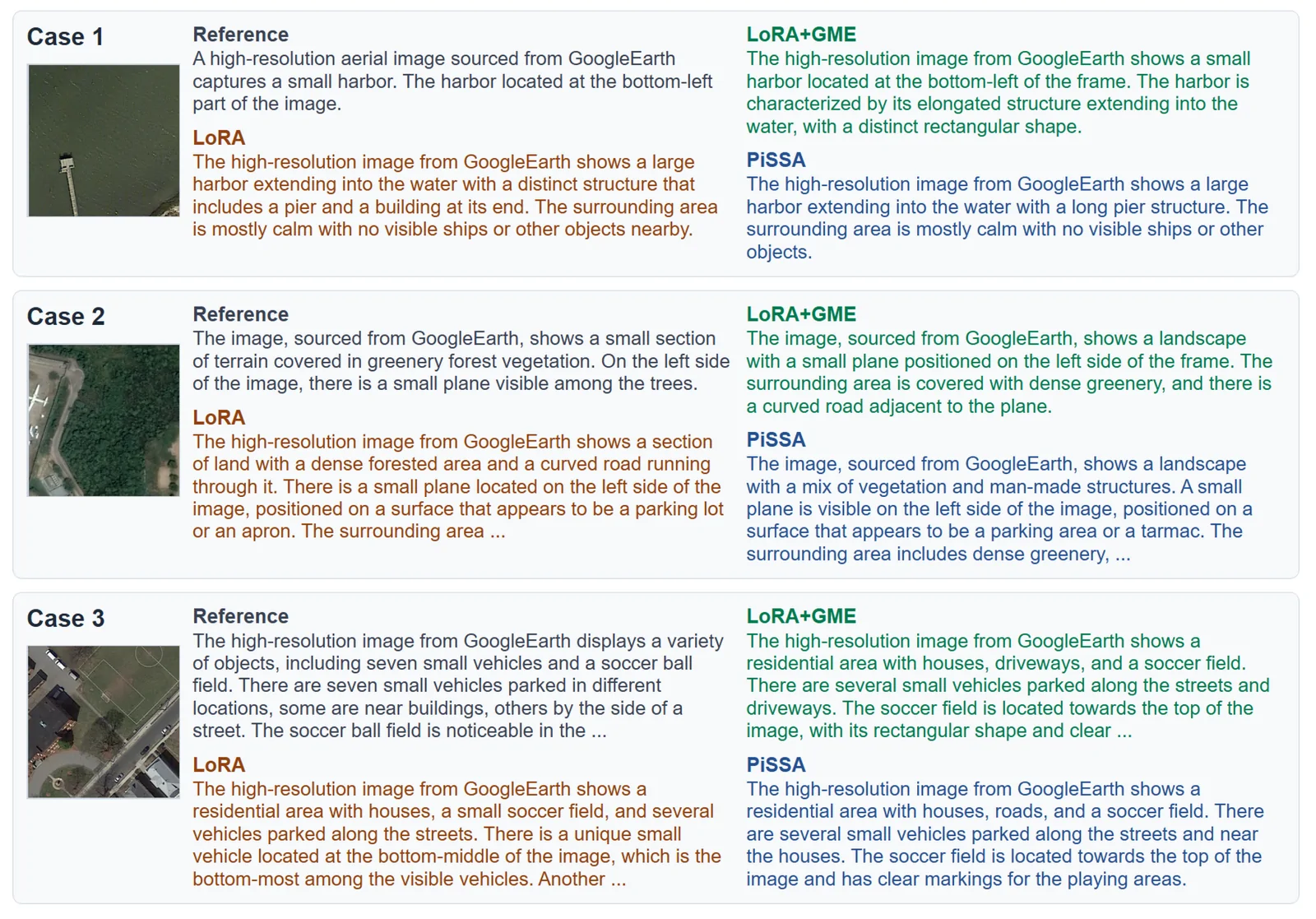
Qualitative VRSBench-Caption examples. Compared with standard LoRA, LoRA+GME produces descriptions that are closer to the reference captions for scene elements such as harbor structures, residential layouts, roads, vehicles, and sports field regions while reducing overly generic or spatially inaccurate descriptions.

**Figure 3:**
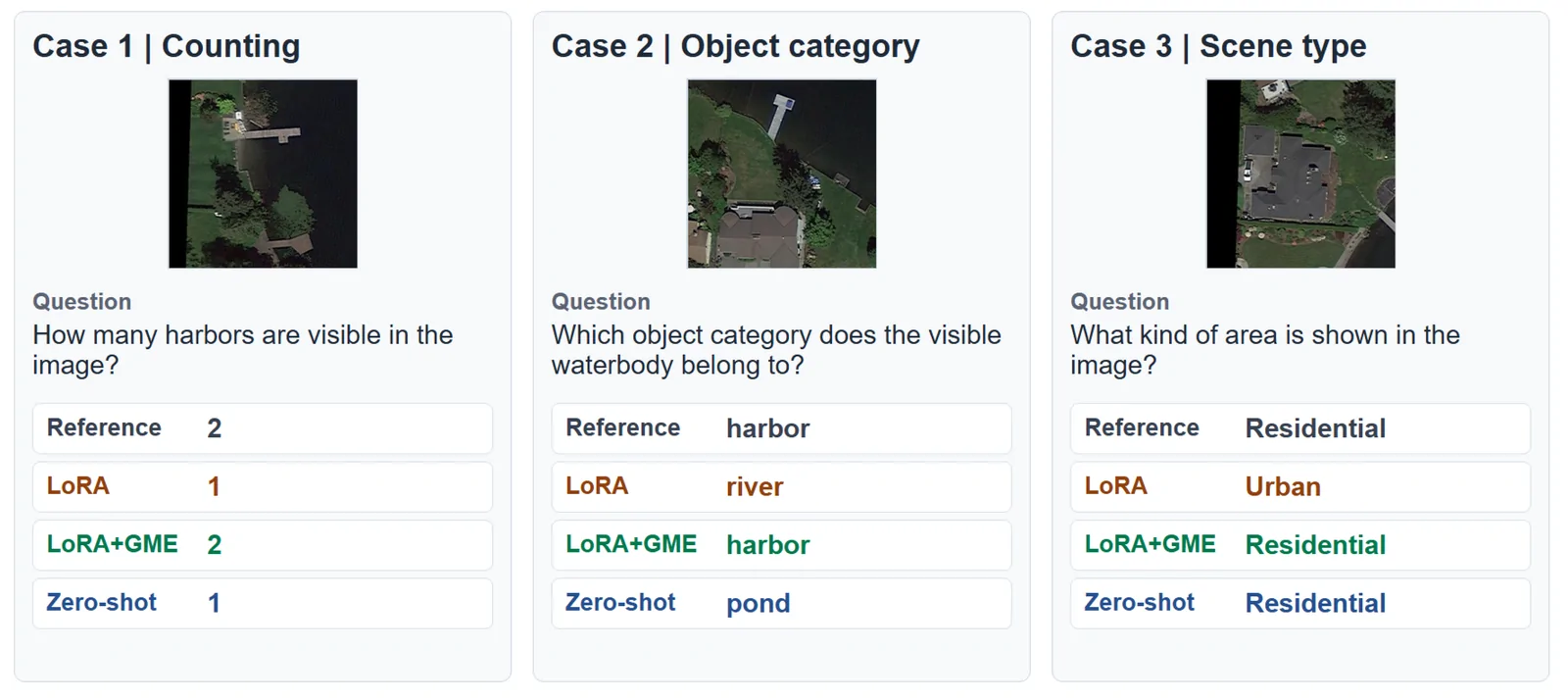
Qualitative VRSBench-VQA examples. GME mainly improves prediction consistency for short-answer questions involving object counting, ground–object category recognition, and scene-type judgment.

**Figure 4:**
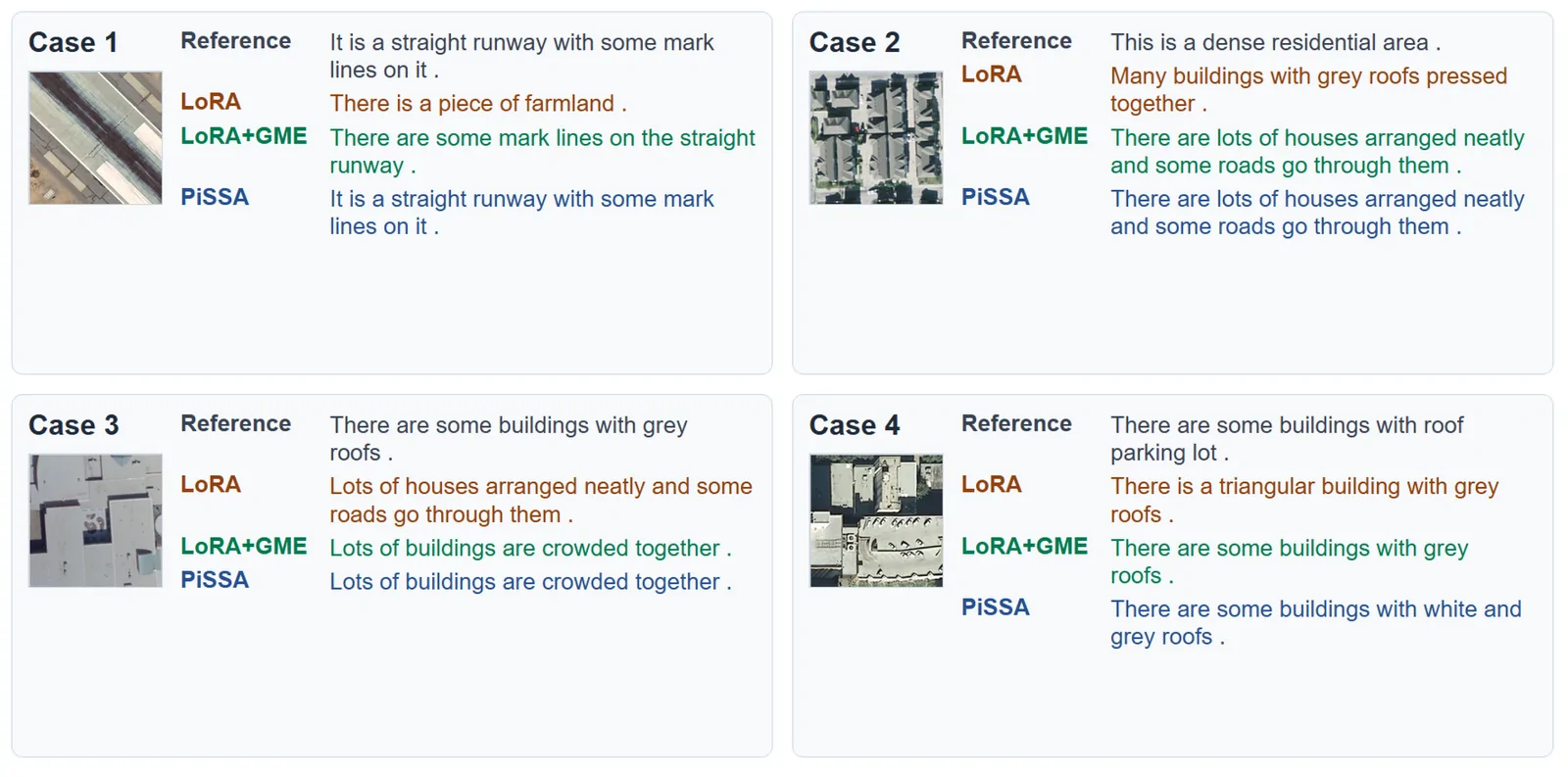
Qualitative UCM-Caption cross-dataset examples. The cases illustrate the effect of GME initialization on generated captions for runway, residential, and building roof scenes under a different captioning distribution.

In addition to quantitative metrics, we present qualitative remote sensing examples that compare VQA and captioning outputs across initialization strategies. Figure 2 shows VRSBench-Caption descriptions of harbor structures, residential layouts, roads, vehicles, and sports field regions. Figure 3 covers object counting, ground–object category recognition, and scene-type judgment on VRSBench-VQA. Figure 4 presents UCM-Caption examples of artificial structures, road textures, and residential scenes under a different captioning distribution. These examples do not replace the statistical results. Instead, they show that the measured changes correspond to differences in remote sensing image semantics rather than numerical fluctuations alone.

### 4.11. Experimental Discussion

The effect of GME-Init can be understood from three perspectives. On GLUE, GME improves the five-task average over standard LoRA at every evaluated rank and achieves the highest average among the compared initialization methods. At rank 8, it also exceeds ConsNoTrainLoRA, CorDA, and LoRA-GA under the same protocol. These results suggest that input-induced output moments can provide a useful basis for asymmetric LoRA initialization in text information processing tasks. The component ablation shows that non-zero *B* initialization and asymmetric Gamma sampling are beneficial; however, the larger gain comes from output-moment calibration, particularly the joint use of variance, skewness, and the full effective response matrix. Improvements are especially visible on RTE and CoLA, which are both relatively small or linguistically sensitive tasks. This pattern is consistent with the role of output variance and skewness in early optimization.

GME-Init also transfers to the evaluated remote sensing vision–language tasks. On VRSBench-VQA, GME simultaneously improves Exact Match, Contains Match, and Token F1. The gains appear in exact short-answer matching, keyword inclusion, and word-level overlap rather than in relaxed matching alone. GME also achieves the highest mean on all three VQA metrics in the direct comparison with ConsNoTrainLoRA and CorDA. On VRSBench-Caption and UCM-Caption, it improves the CIDEr, ROUGE-L, and METEOR-lite scores of standard LoRA. Its mean CIDEr score also exceeds that of ConsNoTrainLoRA in both the 1k and 5k VRSBench-Caption settings. These results suggest that adapting initialization to task-specific skewed representations can improve the expression of scene semantics in the evaluated multimodal generation tasks.

GME-Init is also complementary to existing LoRA variants. PiSSA and OLoRA use pretrained weight decomposition or orthogonal subspace construction, whereas GME preserves the LoRA structure and training configuration and changes only the initialization. The AdaLoRA and DoRA experiments further show that GME can be combined with low-rank budget allocation and weight reparameterization. We do not present GME as a replacement for every initialization or PEFT method, instead emphasizing its plug-in compatibility as an output-moment-driven initialization module.

## 5. Discussion and Limitations

This paper focuses on LoRA initialization rather than a new pretrained backbone or adapter architecture. GME-Init uses a small calibration set to estimate layer-output variance and skewness. The quality of these estimates may depend on how well the calibration samples represent the task input distribution. We use fixed calibration settings for both GLUE and remote sensing, and the remote sensing captioning results remain stable with 32–512 calibration samples. Nevertheless, smaller datasets, longer generation tasks, and more complex reasoning tasks may require more systematic sample-selection strategies. GME-Init also initializes *B* with a tiny non-zero Gaussian distribution so that the asymmetric initialization of *A* can affect layer outputs. Although this perturbation is controlled, its optimal scale may depend on the model size and task.

The coordinated probing strategy estimates an effective response at each layer rather than the full block Jacobian across all adapters. Consequently, it captures the propagated influence of upstream perturbations at downstream observation layers, but it does not explicitly disentangle the contribution of every individual adapter. The resulting layer-wise correction is therefore an efficient reduced-order approximation to the full cross-layer calibration problem. Future work may investigate layer-isolated probing or structured block-Jacobian estimation to model cross-layer interactions more explicitly.

The evaluated model architectures and tasks are also limited. Our experiments use two substantially different backbones: RoBERTa-base for encoder-based text understanding, and Qwen2.5-VL-3B-Instruct for multimodal generation. However, they do not cover larger text-only decoder models, additional language model families, or larger vision–language backbones. Therefore, while the results support transfer across the evaluated text and multimodal settings, broader generalizability across model scales and architectures remains to be established. We also did not evaluate additional information extraction and generation tasks, remote sensing grounding and detection-oriented tasks, or multi-sensor scenarios. The probe hyperparameters remained fixed across all reported tasks and ranks; a systematic sensitivity analysis of probe scale, probe asymmetry, LoRA rank, and backbone choice remains important. Future work will study calibration sample selection, combinations with weight decomposition methods such as PiSSA, visual tower adaptation, and evaluation on larger and more diverse model families and information processing tasks.

## 6. Conclusions

This paper studies the role of LoRA initialization in parameter-efficient fine-tuning. To address the issue that standard LoRA initializes *A* with a symmetric distribution such as Gaussian or Kaiming initialization, initializes *B* to zero, and does not reflect the effect of the input feature distribution on initialization, we propose Gamma-Moment Equalization Initialization (GME-Init). GME-Init transforms LoRA initialization from fixed symmetric sampling into data-aware asymmetric initialization based on output-moment calibration. By estimating target-layer variance and skewness, measuring effective layer-wise responses under coordinated global probes, computing approximate initialization corrections, and initializing the low-rank branch with a centered Gamma distribution and a tiny non-zero Gaussian distribution, GME-Init improves the statistical match between initial adapter weights and the current data distribution while keeping the perturbation magnitude controlled. Experimental results show that GME-Init consistently improves standard LoRA on GLUE text understanding tasks with RoBERTa-base and achieves the highest five-task average at all three evaluated ranks, including comparisons with PiSSA, OLoRA, and the low-order GME-LO approximation. Applying GME-Init to AdaLoRA and DoRA also yields consistent gains. Further remote sensing VQA and image captioning experiments show that GME-Init improves standard LoRA and selected LoRA-style PEFT methods across VRSBench-VQA, VRSBench-Caption, and UCM-Caption, demonstrating that the initialization strategy transfers across the evaluated text and multimodal information processing settings. Since GME-Init operates only during initialization and does not change the LoRA structure, number of trainable parameters, training budget, or inference cost, it can serve as a simple plug-in data-aware initialization module for enhancing LoRA-style PEFT methods.

## Figures and Tables

**Table 1:** Main results on the GLUE subset. Task values are reported as mean ± standard deviation over five random seeds and converted to a percentage scale; Avg. is the arithmetic mean of the five task means. The best result under each rank is highlighted in bold.

Rank	Method	RTE	MRPC	CoLA	SST-2	STS-B	Avg.
4	LoRA	73.86 ± 1.20	90.45 ± 0.55	59.68 ± 1.00	93.41 ± 0.67	90.32 ± 0.20	81.54
4	OLoRA	73.61 ± 1.18	90.62 ± 0.28	59.81 ± 1.06	93.52 ± 0.74	90.68 ± 0.17	81.65
4	PiSSA	74.39 ± 1.68	90.71 ± 0.90	59.23 ± 1.37	**94.08 ± 0.25**	**90.69 ± 0.18**	81.82
4	GME-LO	**75.99 ± 1.34**	90.77 ± 0.92	59.19 ± 1.11	93.62 ± 0.52	90.35 ± 0.15	81.98
4	GME	75.32 ± 1.46	**90.86 ± 0.44**	**60.91 ± 1.09**	93.83 ± 0.27	90.61 ± 0.16	**82.31**
8	LoRA	74.86 ± 1.89	90.95 ± 0.59	60.83 ± 1.19	93.41 ± 0.44	90.38 ± 0.31	82.09
8	OLoRA	73.49 ± 1.43	91.11 ± 0.33	60.71 ± 0.92	94.11 ± 0.47	**90.84 ± 0.28**	82.05
8	PiSSA	76.43 ± 1.11	90.51 ± 0.51	61.37 ± 0.80	93.78 ± 0.43	90.70 ± 0.15	82.56
8	GME-LO	73.74 ± 2.76	90.97 ± 0.52	61.52 ± 1.86	**94.21 ± 0.21**	90.67 ± 0.19	82.22
8	GME	**76.89 ± 1.30**	**91.64 ± 0.37**	**62.77 ± 1.58**	94.02 ± 0.44	90.63 ± 0.07	**83.19**
16	LoRA	74.97 ± 1.93	91.45 ± 0.88	60.87 ± 1.71	93.67 ± 0.66	90.78 ± 0.15	82.35
16	OLoRA	73.81 ± 2.09	91.90 ± 0.38	60.82 ± 1.35	93.97 ± 0.60	90.47 ± 0.21	82.19
16	PiSSA	75.47 ± 1.62	91.20 ± 0.45	61.81 ± 1.87	93.85 ± 0.28	90.85 ± 0.18	82.64
16	GME-LO	74.66 ± 1.69	91.87 ± 1.67	61.67 ± 1.44	93.81 ± 0.33	90.60 ± 0.13	82.52
16	GME	**77.58 ± 1.33**	**92.13 ± 0.39**	**62.31 ± 1.03**	**94.73 ± 0.29**	**90.95 ± 0.14**	**83.54**

**Table 2:** Comparison with related data-aware LoRA initialization methods on the GLUE subset using RoBERTa-base at rank r=8. Standard LoRA is included as the reference baseline. Task values are reported as mean ± standard deviation over five random seeds and converted to a percentage scale; Avg. is the arithmetical mean of the five task means.

Method	RTE	MRPC	CoLA	SST-2	STS-B	Avg.
LoRA	74.86 ± 1.89	90.95 ± 0.59	60.83 ± 1.19	93.41 ± 0.44	90.38 ± 0.31	82.09
ConsNoTrainLoRA	75.02 ± 1.48	91.72 ± 0.49	60.42 ± 1.11	93.99 ± 0.32	**90.79 ± 0.14**	82.39
CorDA	75.31 ± 0.98	91.65 ± 0.36	60.21 ± 1.67	**94.22 ± 0.42**	90.76 ± 0.12	82.43
LoRA-GA	75.09 ± 0.68	**91.79 ± 0.50**	59.71 ± 2.15	93.83 ± 0.48	88.54 ± 1.14	81.79
**GME**	**76.89 ± 1.30**	91.64 ± 0.37	**62.77 ± 1.58**	94.02 ± 0.44	90.63 ± 0.07	**83.19**

**Table 3:** Plug-in comparison of GME-Init with AdaLoRA and DoRA. Task values are reported as mean ± standard deviation over five random seeds and converted to a percentage scale; Avg. is the arithmetical mean of the five task means.

Method	RTE	MRPC	CoLA	SST-2	STS-B	Avg.
DoRA-DefaultInit	72.98 ± 1.37	91.54 ± 0.59	60.24 ± 1.76	94.46 ± 0.24	90.36 ± 0.20	81.92
DoRA-GMEInit	**73.80 ± 1.27**	**91.70 ± 0.44**	**61.13 ± 1.24**	**94.60 ± 0.13**	**90.40 ± 0.09**	**82.33**
AdaLoRA-DefaultInit	79.10 ± 2.12	91.28 ± 0.83	60.93 ± 0.90	94.07 ± 0.37	90.91 ± 0.36	83.26
AdaLoRA-GMEInit	**80.18 ± 1.27**	**91.87 ± 0.40**	**62.66 ± 1.06**	**94.25 ± 0.30**	**90.98 ± 0.28**	**83.99**

**Table 4:** Component ablation of GME-Init on the GLUE subset with RoBERTa-base at rank r=8; Avg. is the arithmetical mean of the five task means.

Method	Non-Zero *B*	*A* Distribution	Variance Calib.	Skewness Calib.	Response Matrix	GLUE Avg.
Standard LoRA	×	Gaussian/Kaiming	×	×	×	82.09
Non-zero *B* only	✓	Gaussian/Kaiming	×	×	×	82.55
Gamma asymmetric init	✓	Centered Gamma	×	×	×	82.51
Variance-only calibration	✓	Centered Gamma	✓	×	Partial/diagonal	82.84
Skewness-only calibration	✓	Centered Gamma	×	✓	Partial/diagonal	82.79
Full GME	✓	Centered Gamma	✓	✓	Full matrix	**83.19**

**Table 5:** Main results on VRSBench-VQA. All values are mean ± standard deviation over five random seeds and are reported in the original scale produced by the evaluation script.

Method	Exact Match	Contains Match	Token F1
Zero-shot	0.4090 ± 0.0000	0.4810 ± 0.0000	0.4279 ± 0.0000
LoRA	0.5550 ± 0.0028	0.5975 ± 0.0064	0.5668 ± 0.0033
LoRA + GME	**0.5605 ± 0.0046**	**0.6030 ± 0.0096**	**0.5747 ± 0.0089**
DoRA	0.5555 ± 0.0021	0.5960 ± 0.0057	0.5668 ± 0.0023
DoRA + GME	0.5575 ± 0.0011	0.5990 ± 0.0042	0.5696 ± 0.0027
LoRA + PiSSA	0.5095 ± 0.0076	0.5930 ± 0.0054	0.5230 ± 0.0096
ConsNoTrainLoRA	0.5575 ± 0.0021	0.5995 ± 0.0049	0.5706 ± 0.0032
CorDA	0.4385 ± 0.0092	0.4975 ± 0.0078	0.4598 ± 0.0155

**Table 6:** Main results on VRSBench-Caption. All values are mean ± standard deviation over five random seeds and are reported in the original scale produced by the evaluation script; the best CIDEr result under each training size is highlighted in bold.

Train Samples	Method	BLEU-4	ROUGE-L	METEOR-Lite	CIDEr
0	Zero-shot	0.0421 ± 0.0000	0.2290 ± 0.0000	0.3345 ± 0.0000	0.0593 ± 0.0000
1k	LoRA	0.1367 ± 0.0001	0.3577 ± 0.0003	0.4724 ± 0.0000	0.1743 ± 0.0038
1k	LoRA + GME	0.1365 ± 0.0001	0.3594 ± 0.0006	0.4728 ± 0.0003	0.1797 ± 0.0030
1k	DoRA	0.1369 ± 0.0006	0.3574 ± 0.0010	0.4722 ± 0.0002	0.1734 ± 0.0018
1k	DoRA + GME	0.1370 ± 0.0002	0.3604 ± 0.0002	0.4742 ± 0.0003	0.1781 ± 0.0036
1k	LoRA + PiSSA	0.1358 ± 0.0026	0.3619 ± 0.0024	0.4715 ± 0.0003	**0.1832 ± 0.0005**
1k	ConsNoTrainLoRA	0.1356 ± 0.0030	0.3591 ± 0.0027	0.4760 ± 0.0026	0.1764 ± 0.0064
5k	LoRA	0.1425 ± 0.0025	0.3674 ± 0.0023	0.4808 ± 0.0012	0.1861 ± 0.0027
5k	LoRA + GME	0.1440 ± 0.0031	0.3677 ± 0.0026	0.4824 ± 0.0014	**0.1959 ± 0.0061**
5k	LoRA + PiSSA	0.1418 ± 0.0103	0.3663 ± 0.0099	0.4812 ± 0.0110	0.1872 ± 0.0303
5k	ConsNoTrainLoRA	0.1392 ± 0.0029	0.3657 ± 0.0020	0.4829 ± 0.0031	0.1830 ± 0.0084

**Table 7:** Cross-dataset results on UCM-Caption. All values are mean ± standard deviation over five random seeds and are reported in the original scale produced by the evaluation script.

Method	BLEU-4	ROUGE-L	METEOR-Lite	CIDEr
LoRA	0.7255 ± 0.0064	0.8189 ± 0.0131	0.8255 ± 0.0129	3.4732 ± 0.0720
LoRA + GME	0.7334 ± 0.0066	0.8253 ± 0.0106	0.8323 ± 0.0102	3.5667 ± 0.0601
LoRA + PiSSA	**0.7352 ± 0.0089**	**0.8297 ± 0.0010**	**0.8350 ± 0.0003**	**3.5844 ± 0.0017**

**Table 8:** Target module ablation on VRSBench-VQA. All values are mean ± standard deviation over five random seeds.

Method	Exact Match	Contains Match	Token F1
Full target set/LoRA	0.5550 ± 0.0028	0.5975 ± 0.0064	0.5668 ± 0.0033
Full target set/LoRA + GME	**0.5605 ± 0.0046**	**0.6030 ± 0.0096**	**0.5747 ± 0.0089**
Attention q/k/v/o/LoRA	0.5005 ± 0.0064	0.5500 ± 0.0071	0.5099 ± 0.0072
Attention q/k/v/o/LoRA + GME	0.5035 ± 0.0049	0.5535 ± 0.0064	0.5142 ± 0.0051
Attention q/v/LoRA	0.4770 ± 0.0028	0.5345 ± 0.0007	0.4900 ± 0.0004
Attention q/v/LoRA + GME	0.4785 ± 0.0007	0.5355 ± 0.0007	0.4911 ± 0.0002

**Table 9:** Calibration sample ablation on VRSBench-Caption. All values are mean ± standard deviation over five random seeds. Higher values indicate better captioning performance.

Method	BLEU-4	ROUGE-L	METEOR-Lite	CIDEr
32 calibration samples	0.1439 ± 0.0067	0.3680 ± 0.0051	**0.4831 ± 0.0035**	0.1946 ± 0.0054
128 calibration samples	**0.1440 ± 0.0031**	0.3677 ± 0.0026	0.4824 ± 0.0014	**0.1959 ± 0.0061**
512 calibration samples	**0.1440 ± 0.0040**	**0.3686 ± 0.0031**	0.4828 ± 0.0016	0.1916 ± 0.0074

**Table 10:** Output-moment mismatch before and immediately after GME-Init. Lower mismatch is better; Reduction denotes the relative decrease from Before to After. Values retain the native moment scale of each model, task, and training setting; therefore, absolute mismatches should be compared within each row rather than across the GLUE and remote sensing groups.

	Variance Mismatch			Skewness Mismatch		
Task/Setting	Before	After	Reduction	Before	After	Reduction
*GLUE text understanding*						
RTE	0.055435	0.000437	99.21%	0.757684	0.229624	69.69%
MRPC	0.053932	0.000934	98.27%	0.533974	0.080251	84.97%
CoLA	0.061795	0.000619	99.00%	0.074369	0.040440	45.62%
SST-2	0.061845	0.005748	90.71%	0.010632	0.003907	63.25%
STS-B	0.057213	0.000772	98.65%	0.465664	0.049421	89.39%
*Remote sensing vision-language understanding*						
VRSBench-VQA	7.892599	0.216719	97.25%	31.614911	4.067983	87.13%
VRSBench-Caption (1k)	8.237132	0.153738	98.13%	33.889950	12.656412	62.65%
VRSBench-Caption (5k)	8.311804	0.462392	94.44%	35.425013	12.434558	64.90%
UCM-Caption	14.823864	1.550221	89.54%	20.625402	3.567150	82.71%

## Data Availability

The datasets used in this study are publicly available from their original sources.

## Abbreviations

The following abbreviations are used in this manuscript:

CIDEr	Consensus-based Image Description Evaluation
GME	Gamma-Moment Equalization
LoRA	Low-Rank Adaptation
LVLM	Large Vision–Language Model
PEFT	Parameter-Efficient Fine-Tuning
VLM	Vision–Language Model
VQA	Visual Question Answering
